# Cytotoxic Activity of Inositol Angelates and Tirucallane-Type Alkaloids from *Amoora Dasyclada*

**DOI:** 10.3390/molecules25051222

**Published:** 2020-03-09

**Authors:** Sheng-Xiang Yang, Cheng-Gang Song, Yi Kuang, Bing Liu, Yan-Xin Zhang, Ming-Zhe Zhang, Chun-Ying Zhang, Gang Ding, Jian-Chun Qin

**Affiliations:** 1 Zhejiang Provincial Key Laboratory of Chemical Utilization of Forestry Biomass, Zhejiang A&F University, Lin’an 311300, Zhejiang, China; shengxiangyang2000@163.com (S.-X.Y.); jlu_bio@163.com (Y.K.); 2 College of Plant Science, Jilin University, Xi’an Road No. 5333, Changchun 130062, Jilin, China; songcg18@mail.jlu.edu.cn (C.-G.S.); liubing18@mail.jlu.edu.cn (B.L.); zhangyx17@mail.jlu.edu.cn (Y.-X.Z.); mzzhang@jlu.edu.cn (M.-Z.Z.); 3 The clinical medicine department of Changchun Medical College, Jilin Road No. 6177, Changchun 130013, Jilin, China; 15754305325@163.com; 4 Institute of Medicinal Plant Department, Chinese Academy of medical Sciences and Peking Union Medical College, Beijing 100193, China

**Keywords:** inositol angelate, tirucallane alkaloid, *Amoora dasyclada*, cytotoxicity

## Abstract

Three new inositol angelate compounds (**1–3**) and two new tirucallane-type alkaloids (**4** and **5**) were isolated from the *Amoora dasyclada*, and their structures were established mainly by means of combination of 1D and 2D nuclear magnetic resonance and HR-ESI-MS. Based on cytotoxicity testing, compounds **4** and **5** exhibited significant cytotoxic activity against human cancer cell line HepG2 with IC_50_ value at 8.4 and 13.2 μM. In addition, compounds **4** and **5** also showed remarkable growth inhibitory activity to *Artemia salina* larvae.

## 1. Introduction

**Figure 1:**
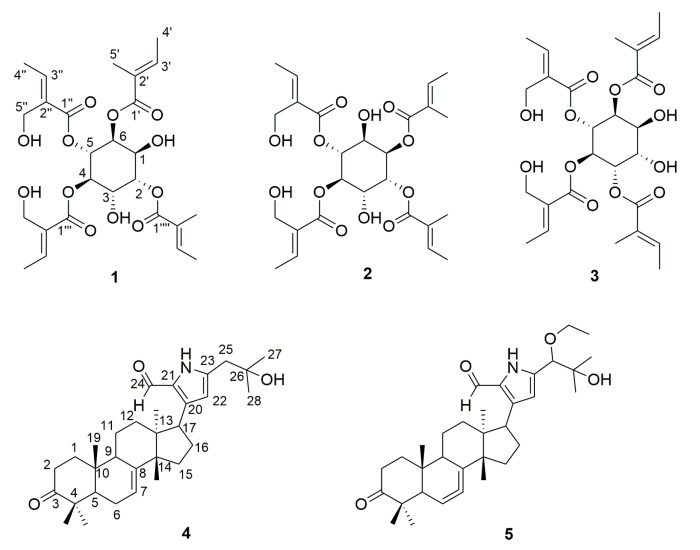
Compounds **1**–**5** isolated from *Amoora dasyclada*.

The Meliaceae family comprises about 1400 species, mainly distributed in tropical and subtropical areas, with 62 among them being found in China. Plants of this family are rich sources of structurally diverse and biologically significant limonoids [1,2]. Some vegetative storage proteins in Meliaceae were investigated by Tian et al. [3]. *Amoora dasyclada*, a member of the Meliaceae family, yields varied kinds of terpenoids, including diterpenoid, triterpenoid and tetraterpenoid, with anticancer and cytotoxic activities [4,5,6,7,8,9,10,11,12,13]. We firstly isolated three new inositol angelates and two alkaloids (compounds **1**–**5**, Figure 1) which have not been investigated previously in *Amoora dasyclada*. Angelate compounds were exhibited to have antitumor activity: 3-ingenyl angelate has significant inhibitory effects on skin cancer [14]; decursinol angelate caused a significant increase in the life span and a significant decrease in the tumor weight and volume of mice inoculated with Sarcoma-180 tumor cells [15]. Tirucallane derivatives with a pyrrole ring from Meliaceae family showed obvious cytotoxicity against five human cancer cell lines [7]. Compounds **1**–**5** were identified by 1D and 2D NMR method and HR-ESI-MS and structurally compared with similar compounds found previously.

## 2. Results and Discussion

### 2.1. Identification of Compounds

Compounds **1**–**3** were identified as inositol angelates. According to the structure of the previously isolated compound from *Inula cappa*, it is similar to *cis*-1,2,3,5-*trans*-4,6-inositol-2,3,6-triangelate and l-inositol-1,2,3,5-tetraangelate [16,17]. The inositol angelates isolated from *Amoora dasyclada* have hydroxyl groups attached to two of its side chains. The chemical shifts of carbon and hydrogen at the same position are changed due to the strong electron-withdrawing ability of the hydroxyl group.

**Figure 2:**
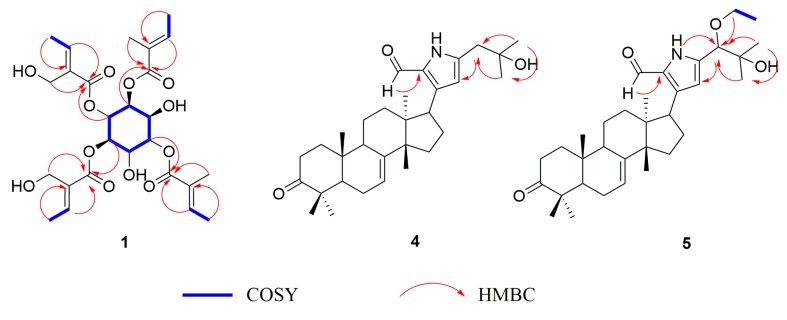
^1^H-^1^H COSY and HMBC correlations of **1**, **4** and **5**.

Molecular formula of compound **1** was determined to be C_26_H_36_O_12_ on the basis of its HR-ESI-MS data (*m/z* 563.2109 [M + Na]^+^, calcd for C_26_H_36_NaO_12_: 563.2106), requiring nine degrees of unsaturation. The IR and UV spectrum exhibited absorption bands for -OH, C=O, and C=C groups. Analysis of ^13^C-NMR and DEPT data indicate 26 carbon resonances including six methyl, two methylene, ten methine and eight quaternary carbons (Table 1). ^1^H-NMR data show four hydroxyl and four carbon–carbon double-bonds, which revealed four ester groups and four olefinic bonds. Furthermore, the remaining unsaturation demonstrates a six-carbon skeleton established by COSY data. Two meta hydroxyls were elucidated by HMBC correlations between *δ*_C_ 66.3 (1-OH), *δ*_C_ 66.4 (3-OH) and *δ*_C_ 72.5 (C-2); moreover, relationships between 2-H and 1′-C, 4-H and 1′′-C, 5-H and 1′′′-C, 6-H and 1′′′′-C revealed the four esterification substituent groups of inositol. According to the data comparison, the overall structure of compound **1** is almost similar to the known compound angelate from *Inula cappa*. The difference is that HMBC correlations from *δ*_C_ 4.01 (5′′-2H) and *δ*_H_ 4.06/4.09 (5′′′-2H) to *δ*_C_ 165.9 (C-1′′, C-1′′′) show the interaction from extra hydroxyls to carbonyl, indicated that two side chains on the carbon ring each connected to a hydroxyl group, which does not exist in the previously identified compound (Figure 2).

Compounds **2** and **3** are isomers of compound **1**; their chemical shifts are basically the same. The differences between them are embodied in the relative position of two hydroxyls on the ring (adjacent in compound **2** and para in compound **3**) which were proved by HMBC and COSY data. The relative stereochemistry of **1**–**3** was determined by circular dichroism spectrum recorded in MeOH. According to the ECD data (Figure S32, supplementary information) and quantum-chemical calculation using time-dependent density functional theory, the absolute configuration of compound **3** is indicated to be (1*S*,2*S*,3*R*,4*R*,5*S*,6*S*). Considering the location of hydroxyls and comparing to the CD data of **3**, we establish the absolute configurations of compound **1** (1*S*,2*S*,3*S*,4*R*,5*R*,6*S*) and **2** (1*R*,2*R*,3*S*,4*R*,5*R*,6*S*). 

Compound **4** and **5** were identified to be tirucallane-type alkaloids; common to these alkaloids is the attachment of a pyrrole ring having small groups such as aldehyde or alkyl groups to the C-17 of the main structure of the tirucallane [7]. Structures of compound **4** and **5** were elucidated based on known scaffold data of compounds from *Dysoxylum laxiracemosum*.

Compound **4** was determined to be C_3__1_H_45_NO_3_ on the basis of HR-ESI-MS data (*m/z* 480.3475 [M + H]^+^, calcd for C_31_H_46_NO_3_: 480.3478), requiring nineteen degrees of unsaturation. The *δ*_H_ 7.26 and 9.48 indicate an extra hydroxyl and aldehyde. Further analysis by the correlations from *δ*_H_ 7.26 to *δ*_C_ 129.9 (C-21) and 138.6 (C-20) confirmed the aldehyde attached to the C-21 of pyrrole ring. HMBC correlations from *δ*_H_ 1.25, 1.24 (6H, H-27/28) to *δ*_C_ 70.9 (C-26) and 41.3 (C-25) indicated an isopropyl group attached to C-25.

The molecular formula of compound **5** was C_3__3_H_49_NO_4_ (*m/z* 524.3731 [M + H]^+^, calcd for C_33_H_50_NO_4_: 524.3740), whose structure was basically the same as compound **4** but with one more oxygen atom than compound **4** from the chemical formula. An extra ethoxy was pointed to from *δ*_H_ 3.33 and 1.17, while the obvious signal in HMBC data between *δ*_H_ 3.33 and *δ*_C_ 83.6 (C-25) showed an oxyethyl attached on C-25.

### 2.2. Bioactive Assay

**Figure 3:**
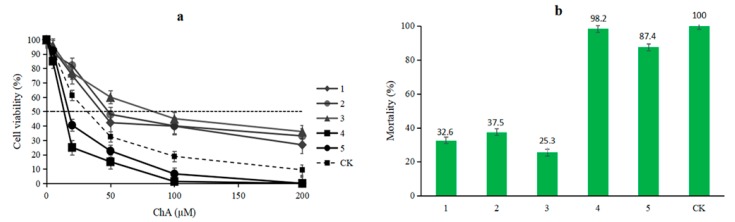
Bioactive assay of compounds **1**–**5: (a)** Cell viability inhibition of HepG2 cell, CK: camptothecin; **(b)** mortality rates to *Artemia salina*, CK: actinomycin D.

Compounds **1**–**5** were evaluated for their cytotoxicity against human cancer cell line hepatoblastoma (HepG2) by the MTT method. Compounds **1**–**3** showed moderate toxicity, with IC_50_ values of 40.3, 48.9 and > 50 μM, respectively. Compounds **4** and **5** exhibited significant activity with the IC_50_ values of 8.4 and 13.2 μM, respectively, compared to camptothecin (32.3 µM) (Figure 3a). In addition, compounds **1**–**5** were evaluated by brine shrimp bioassay, and the mortality rates to *Artemia salina* were 32.6%, 37.5%, 25.3%, 98.2% and 87.4%, respectively, at a concentration of 10 µg/mL, in contrast to actinomycin D (100%) (Figure 3b).

## 3. Materials and Methods

### 3.1. General Experimental Procedures

Optical rotations were taken on a Perkin-Elmer 241 polarimeter (PerkinElmer, Waltham, MA, USA). The 1D and 2D NMR spectra were measured in acetone-d6 and DMSO-*d_6_* (dH 2.09/dC 206.0) on a Bruker 600 spectrometer (^1^H: 500MHz; ^13^C: 125MHz) (Bruker, Rheinstetten, Germany). HR-ESI-MS were obtained using an ESI-MS (Electrospray Ionization Mass Spectrometer) (Waters Synapt G2, Milford, MA, USA). Semipreparative HPLC separation was performed on a Shimadzu LC-6AD instrument packed with a YMC-Pack ODS-A column (5 µm, 250 mm × 10 mm). Column chromatographies (CC) were carried out on Sephadex LH-20 (Amersham Biosciences, Uppsala, Sweden) and silica gel (Qingdao Haiyang Chemical Co., Ltd., Qingdao, China).

### 3.2. Plant Material

*Amoora dasyclada* (How et T. Chen) C. Y. Wu, whole plant collected from Xiao-Meng-Lun, Yunnan province, China in August 2013 was used as plant material.

### 3.3. Extraction and Isolation

The dry branches (16 kg) was smashed directly with ultrasonic extraction by ethanol three times successively, then the extract (820 g) was separated by silica gel CC, eluting with petroleum-ether–acetone step-gradient (100:1, 80:1, 60:1, 40:1, 20:1, 10:1, 5:1, 1:1, 0:1, MeOH) to get the fractions 1–10. Fraction 7 (60:1) was subjected to silica gel (CH_2_Cl_2_–MeOH, 300:1 to 80:1) to yield fraction 7.3 and fraction 7.4. Fraction 7.3 was chromatographed on silica gel (petroleum-ether–EtOAc, 50:1 to 10:1), sephadex LH-20 (CH_2_Cl_2_–MeOH) and silica gel (CH_2_Cl_2_–MeOH, 200:1) to give compound **4**. Fraction 7.4 was subjected to MPLC (speed 20 mL/min, 80% MeOH to 100% MeOH), sephadex LH-20 (CH_2_Cl_2_–MeOH) and silica gel CC (petroleum-ether–EtOAc, 10:1) to give compound **5**. Fraction 10 was applied with silica gel CC (CH_2_Cl_2_–MeOH, 100:1 to 5:1) to obtain fraction 10.2 (50:1) and fraction 10.3 (40:1). Fraction 10.2 was chromatographed on MPLC (70% MeOH), sephadex LH-20 (MeOH) and then silica gel (CH_2_Cl_2_–MeOH, 50:1) to give compound **3**. Fraction 10.3 was chromatographed MPLC (70% MeOH to 40% MeOH). Then, 33% methanol part and 35% methanol part were chromatographed on silica gel CC (CH_2_Cl2–MeOH, 100:1 to 20:1) to give compounds **1** and **2**, respectively.

### 3.4. Cytotoxic Evaluation

Compounds **1**–**5** were evaluated for their cytotoxic activities to HepG2 tumor cell line and the growth inhibitory activity towards brine shrimp by using the colorimetric assay and small modified microtiter-plate method, respectively as previously described in the literature [18,19].

Cytotoxic activities of compounds **1**–**5** were examined with the MTT colorimetric method. The HepG2 cells (2 × 10^4^) were grown in Eagle’ s Minimum Essential Medium, then seeded and attached in 96-well plates for 24 h at 37 °C in 5% CO_2_. Then, the cells were treated with **1**–**5** using different concentrations and incubated for 72 h. Then, the cells were fixed for 1 h at 4 °C with 50% trichloroacetic acid (TCA). Subsequently, the cells were washed with distilled water and dried. Then, 100 μL of SRB was added to each well, and the plate was incubated for 30 min at RT. To remove the excess dye, the cells were washed using 1% acetic acid. Absorption at 540 nm was measured with an ELISA plate reader, and the IC_50_ value was defined as the concentration at which 50% survival of cells was discerned. All experiments were performed in duplicate and were repeated three times.

Brine shrimp toxicity was assayed by small modified microtiter-plate method using brine shrimp *Artemia salina* as a test organism. Briefly, approximately 30 nuclei larvae hatched from eggs of *A. salina* in 0.2 mL of artificial sea water were incubated with a sample (5 mL in DMSO solution) in a deep-well microtiter plate at room temperature. After 24 h, the dead larvae were determined by counting the number of the dead animals in each well under microscope. To each test row, positive sample was accompanied by adding actinomycin D.

## 4. Conclusions

Three new inositol angelate compounds (**1**–**3**) and two new tirucallane-type alkaloids (**4** and **5**) were isolated from the *Amoora dasyclada* and their structures were established. Compounds **4** and **5** exhibited significant cytotoxic activity against human cancer cell line HepG2 with IC_50_ values at 8.4 and 13.2 μM. In addition, compounds **4** and **5** also showed remarkable toxicity to the larvae of *Artemia salina*.

*Amoora dasyclada*, together with *Amoora ouangliensis* and *Amoora stellato-spuamosa*, belongs to the terpenoid-rich genus *Amoora* Roxb. (Meliaceae), which is followed with interest in the studies on the chemicals with antitumor properties [8,9,10,11,12]. Two novel tirucallane triterpenoids (**4** and **5**) with cytotoxicity identified in this research can provide evidence of chemotaxonomy and medicinal value for this species. Additionally, the rarely studied inositol angelates had only been isolated from the Chinese traditional medicine herb *Inula cappa* before, which is an ingredient of a famous Chinese formula against fever, abdominal distention and menoxenia [16,17]. Identification and evaluation of three new inositol angelates (**1**–**3**) from *Amoora dasyclada* in this work suggest the further definition of the roles of *Amoora dasyclada* in phytochemistry research and medicinal application.

## Figures and Tables

**Table 1:** ^1^H- and ^13^C-NMR spectra data of **1–5**.

No.	1		2		3		No.	4		5	
	*δ*_C_	*δ*_H_	*δ*_C_	*δ*_H_	*δ*_C_	*δ*_H_		*δ*_C_	*δ*_H_	*δ*_C_	*δ*_H_
1	66.3	4.00, m	69.4	4.19, m	70	4.34, m	1	38.4	1.97, m	39.6	1.94, m
2	73.7	5.11, dd (4.2, 3.2)	72.9	5.36, dd (9.6, 6.9)	70	4.34, m			1.43, m		1.42, m
3	66.4	4.10, dd (10.2, 3.2)	72.9	5.36, dd (9.6, 6.9)	71.4	5.54, dd (5.5, 3.8)	2	34.8	2.75, m	35.8	2.83, m
4	72.3	5.27, t (10.2, 6.5)	69.4	4.19, m	70.5	5.79, dd (5.5, 3.8)			2.23, m		2.14, m
5	69.4	5.52, t (10.0, 6.5)	70.3	5.42, t (5.5, 3.8)	70.5	5.79, t (10.0, 6.5)	3	216.9		219.1	
6	71.9	4.91, dd (10.5, 2.9)	70.3	5.42, t (5.5, 3.8)	71.4	5.54, dd (5.5, 3.8)	4	47.9		48.8	
1′	166.1		167.6		167		5	52.4	1.89, m	53.9	1.88, m
2′	127.9		131.3		127.7		6	24.4	1.97, m	24.3	1.97, m
									2.05, m		2.05, m
3′	138.3	6.86, brq (7.3)	143.2	7.02, q (7.0)	13.9	6.84, brq (7.2)	7	118.2	5.36, br m	115.4	5.39, br m
4′	14.4	1.83, d (7.3)	14.4	1.89, s	14.5	1.74, d (7.3)	8	145.5		146.9	
5′	12.1	1.83, s	56.3	4.36, d (12.6)	12	1.75, s	9	48.2	2.34, m	49.7	2.38, m
				4.32, d (12.6)			10	35.2		36.4	
1′′	165.9		167.6		166.8	-	11	17.3	1.60, m	18.3	1.63, m
2′′	132.5		131.3		131.3	-	12	30.1	1.39, m	31.3	1.39, m
									1.45, m		1.46, m
3′′	140.7	6.75, q (7.2)	143.2	7.02, q (7.0)	143	6.91, q (7.2)	13	45.2		46.5	
4′′	14.2	1.81, d (7.2)	14.4	1.89, s	14.4	1.84, d (7.2)	14	50.9		52.1	
5′′	54.6	4.06, d (12.0)	56.3	4.36, d (12.6)	56	4.20, d (12.0)	15	34.2	1.66, m	35.8	1.66, m
									1.73, m		1.74, m
		4.09, d (12.0)		4.32, d (12.6)		4.24, d (12.0)	16	28.2	1.97, m	24.9	1.98, m
									2.18, m		2.17, m
1′′′	165.9		166.8		166.8		17	43.3	3.41, dd (9.3, 9.4)	44.4	3.57, dd (9.4, 9.4)
2′′′	132.5		127.5		131.3		18	23.1	0.73, s	23.8	0.76, s
3′′′	140.5	6.61, q (7.2)	140	6.96, brq (7.2)	143	6.91, q (7.2)	19	12.6	1.03, s	13.0	1.07, s
4′′′	14.1	1.76, d (7.2)	12.2	1.86, s	14.4	1.84, d (7.2)	20	138.6		141.4	
5′′′	54.4	4.01, 2H, s	14.7	1.88, s	56	4.20, s	21	130		131.1	
						4.24, s	22	112.4	6.02, s	113.2	6.24, s
1′′′′	166.6		166.8		167		23	138.2		141.0	
2′′′′	127.5		127.5		127.7		24	176.7	9.48, s	179.1	9.49, s
3′′′′	138.2	6.69, brq (7.3)	140	6.96, brq (7.2)	13.9	6.84, dd (10.0, 6.9)	25	41.3	2H, 2.76, s	83.6	4.13, s
4′′′′	14.3	1.70, d (7.2)	12.2	1.86, s	14.5	1.74, d (7.2)	26	70.9		73.5	
5′′′′	11.8	1.65, s	14.7	1.88, s	12	1.75, s	27	29.6	1.25, s	29.8	1.25, s
							28	29.5	1.24, s	29.5	1.23, s
							29	21.5	1.12, s	22.0	1.13, s
							30	25.5	1.04, s	25.0	1.02, s
							31	27.5	1.13, s	28.0	1.18, s
							32			66.5	3.33–3.48, m
							33			15.5	1.17, m

## Supplementary Materials

The supplementary materials are available online.
