# Supplementary material for: Cytotoxic Activity of Inositol Angelates and Tirucallane-Type Alkaloids from Amoora Dasyclada
Source: Molecules. 2020 Mar 9;25(5):1222. doi: 10.3390/molecules25051222 (PMC7179408; doi:10.3390/molecules25051222)
Supplement: Supplementary file 1 [file molecules-25-01222-s001.pdf]

Supporting Information

Cytotoxic Activity of Inositol Angelates and Tirucallane-type Alkaloids from *Amoora dasyclada*

Figure S1. ^{1}H NMR (400 MHz, $CDCl_3$) spectrum of the new compound **1**. 3

Figure S2. ^{13}C NMR (100 MHz, $CDCl_3$) spectrum of the new compound **1**. 4

Figure S3. COSY NMR spectrum of the new compound **1** in $CDCl_3$. 5

Figure S4. HSQC NMR spectrum of the new compound **1** in $CDCl_3$. 6

Figure S5. HMBC NMR spectrum of the new compound **1** in $CDCl_3$. 7

Figure S6. ROESY NMR spectrum of the new compound **1** in $CDCl_3$. 8

Figura S7. Mass spectra of **1**...9
Figure S8. ^{1}H NMR (400 MHz, CDCl3) spectrum of the new compound **2**..............10
Figure S9. ^{13}C NMR (100 MHz, $CDCl_3$) spectrum of the new compound **2**. 11
Figure S10. COSY NMR spectrum of the new compound **2** in $CDCl_3$. 12
Figure S11. HSQC NMR spectrum of the new compound **2** in $CDCl_3$. 13
Figure S12. HMBC NMR spectrum of the new compound **2** in $CDCl_3$. 14
Figure S13. ROESY NMR spectrum of the new compound **2** in $CDCl_3$...................15
Figura S14. Mass spectra of **2.**...16
Figure S15. 1H NMR (400 MHz, CDCl3) spectrum of compound **3**. 17
Figure S16. ^{13}C NMR (100 MHz, CDCl3) spectrum of the compound **3**. 18
Figure S17. COSY NMR spectrum of the new compound **3** in $CDCl_3$. 19
Figure S18. HSQC NMR spectrum of the new compound **3** in $CDCl_3$. 20
Figure S19. HMBC NMR spectrum of the new compound **3** in $CDCl_3$. 21
Figure S20. ROESY NMR spectrum of the new compound **3** in $CDCl_3$...................22
Figura S21. Mass spectra of **3**...23
Figure S22. ^{1}H NMR (400 MHz, CDCl3) spectrum of compound **4.**.........................24
Figure S23. ^{13}C NMR (100 MHz, CDCl3) spectrum of the compound **4**. 25
Figure S24. HSQC NMR spectrum of the new compound **4** in $CDCl_3$. 26
Figure S25. HMBC NMR spectrum of the new compound **4** in $CDCl_3$. 27
Figura S26. Mass spectra of **4**...28
Figure S27. ^{1}H NMR (400 MHz, CDCl3) spectrum of compound **5**.........................29
Figure S28. ^{13}C NMR (100 MHz, CDCl3) spectrum of the compound **5**. 30
Figure S29. HSQC NMR spectrum of the new compound **5** in $CDCl_3$. 31
Figure S30. HMBC NMR spectrum of the new compound **5** in $CDCl_3$. 32
Figura S31. Mass spectra of **5**...33
Figura S32. ECD and UV spectrum of **3**. 34

Figure S1. ^{1}H NMR (400 MHz, $CDCl_3$) spectrum of the new compound **1**.

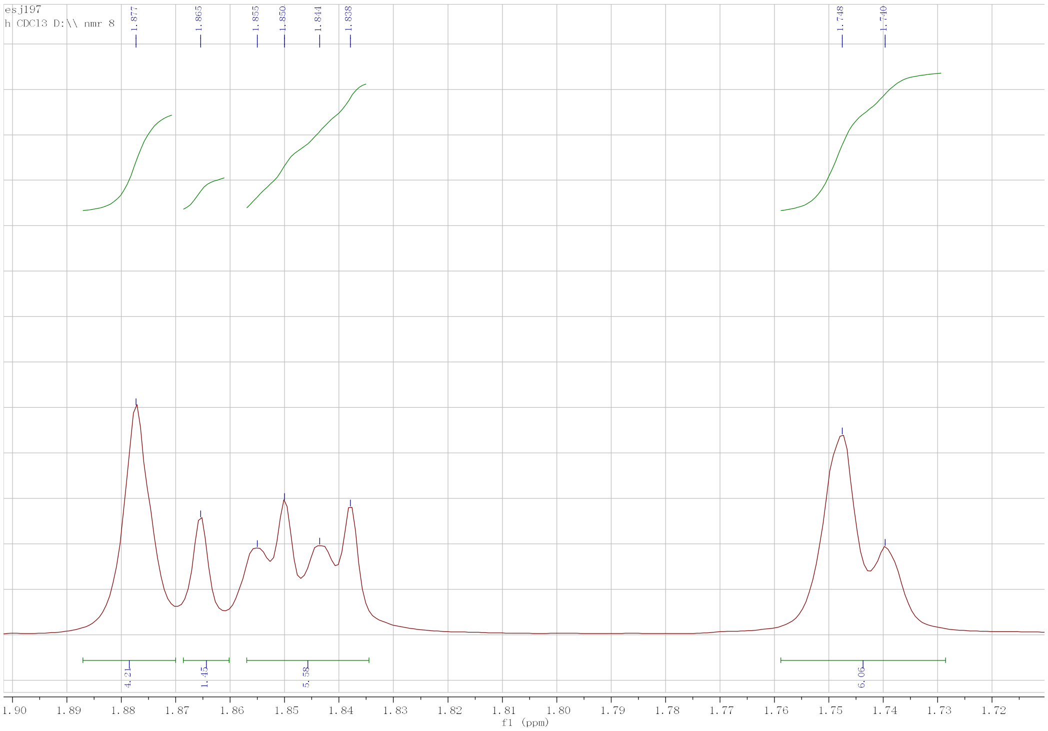

Figure S2. ^{13}C NMR (100 MHz, $CDCl_3$) spectrum of the new compound **1**.

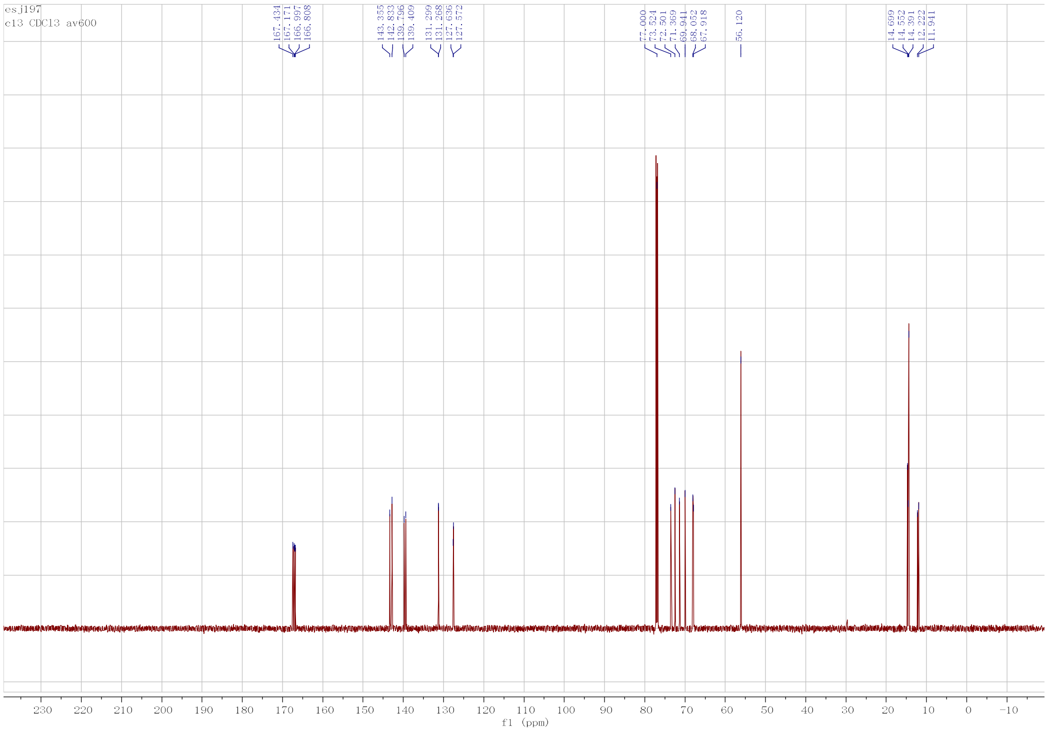

Figure S3. COSY NMR spectrum of the new compound **1** in $CDCl_3$.

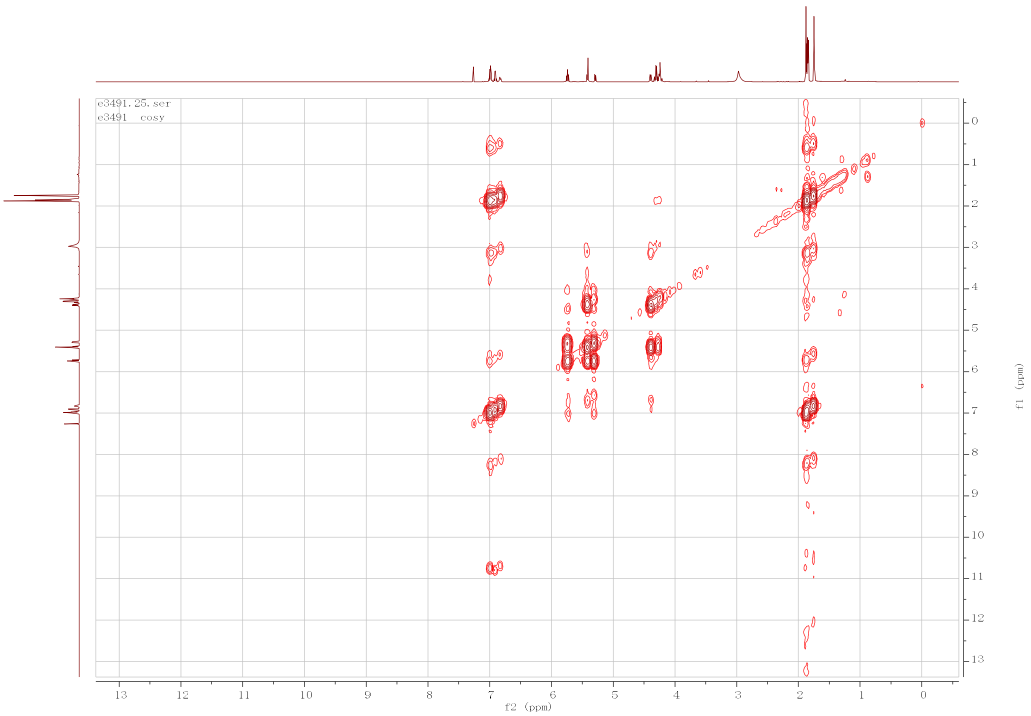

Figure S4. HSQC NMR spectrum of the new compound **1** in $CDCl_3$.

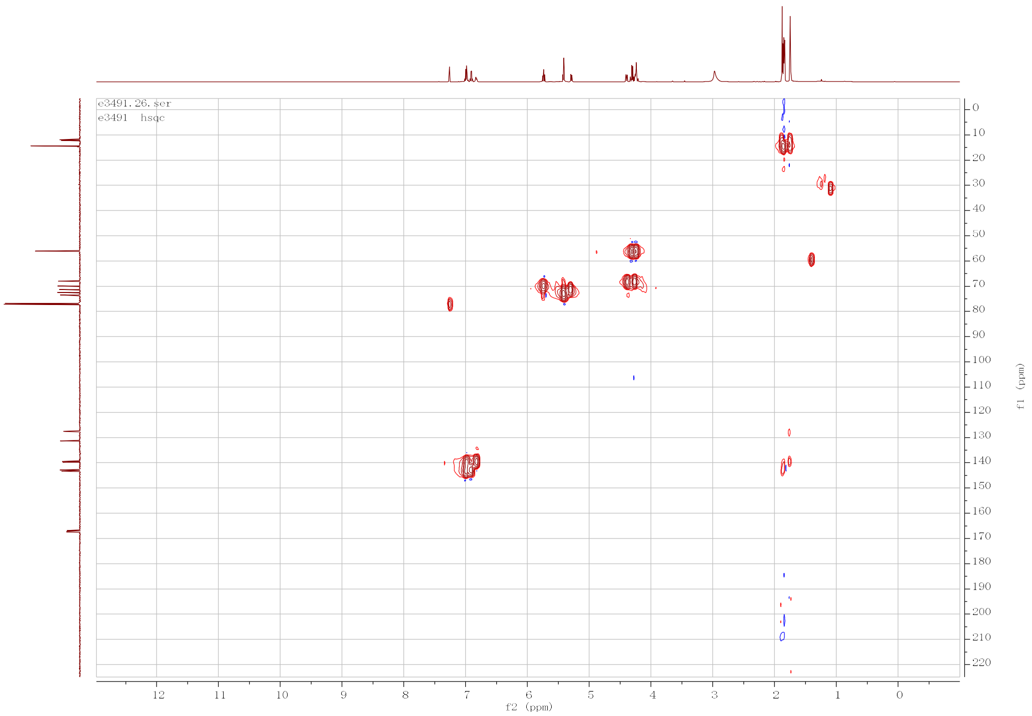

Figure S5. HMBC NMR spectrum of the new compound **1** in $CDCl_3$.

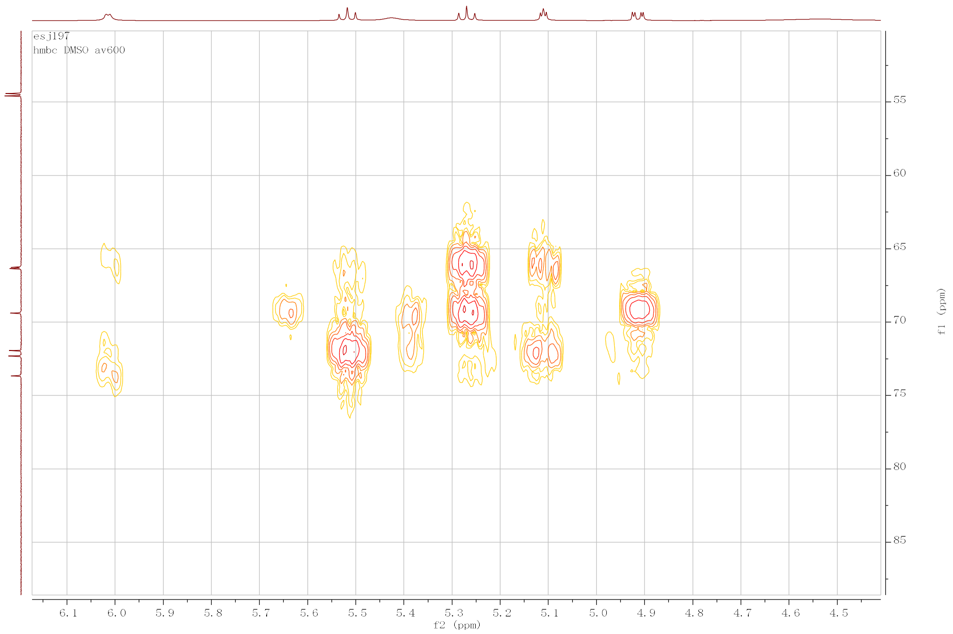

Figure S6. ROESY NMR spectrum of the new compound **1** in $CDCl_3$.

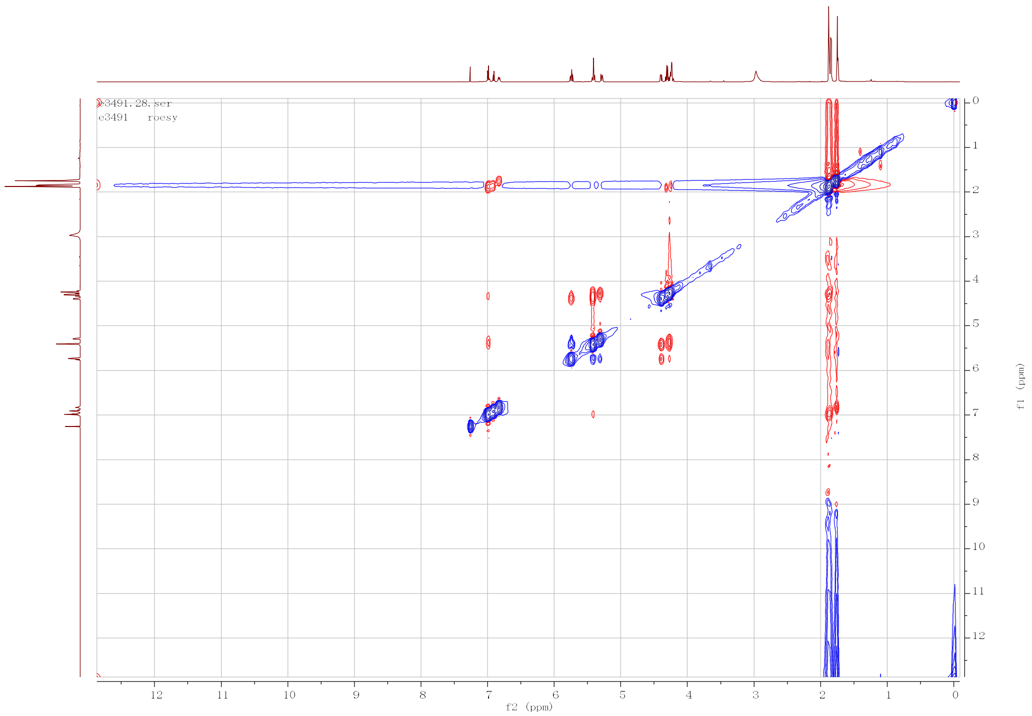

Figura S7. Mass spectra of **1**.

Qualitative Analysis Report

**Data Filename**	151014ESIA8.d	**Sample Name**	Esj197
**Sample Type**	Sample	**Position**	
**Instrument Name**	Agilent G6230 TOF MS	**User Name**	KIB
**Acq Method**	ESI.m	**Acquired Time**	10/13/2015 2:55:20 PM
**IRM Calibration Status**	Success	**DA Method**	ESI.m
**Comment**			

Sample Group **Info.**
Acquisition SW 6200 series TOF/6500 series
Version Q-TOF B.05.01 (B5125.2)

User Spectra

Fragmentor Voltage	Collision Energy	Ionization Mode
200	0	ESI

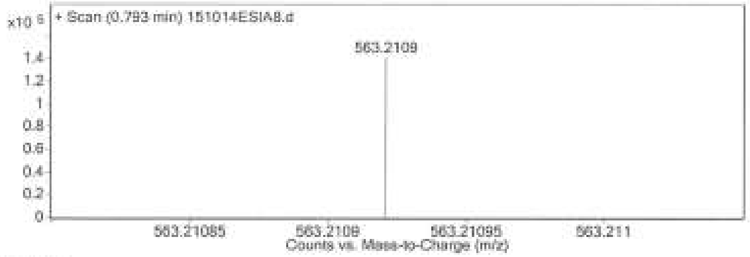

Peak List

m/z	z	Abund	Formula	Ion
121.0509	1	17885.99		
563.2109	1	141288.53	C26 H36 Na O12	M+
564.2138	1	33877.46	C26 H36 Na O12	M+
922.0098	1	23466.64		
1098.4736	1	19254.93		
1103.4312	1	171035.55		
1104.4339	1	92534.48		
1105.4371	1	30705.88		
1643.6477	1	19724.38		
1644.6502	1	17099.18		

Formula Calculator Element Limits

Element	Min	Max
C	0	200
H	0	400
O	8	15
Na	1	1

Formula Calculator Results

Formula	CalculatedMass	CalculatedMz	Mz	Diff. (mDa)	Diff. (ppm)	DBE
C26 H36 Na O12	563.2105	563.2099	563.2109	-0.9	-1.5	8.5000

--- End Of Report ---

Figure S8. ¹H NMR (400 MHz, CDCl3) spectrum of the new compound **2**.

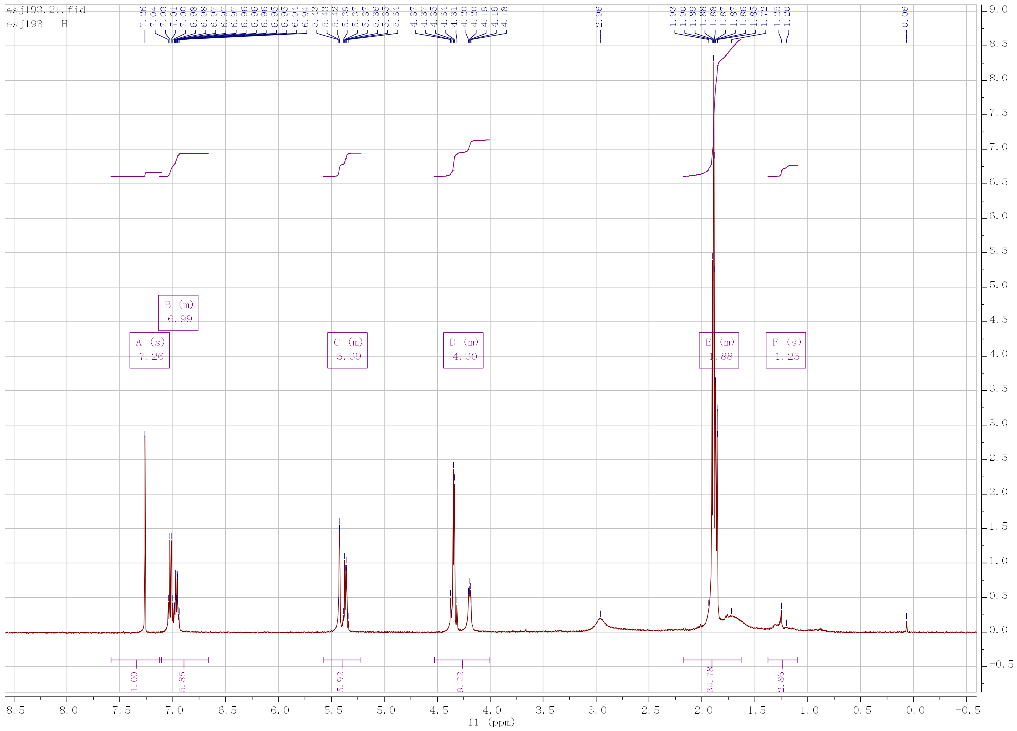

Figure S9. ^{13}C NMR (100 MHz, $CDCl_3$) spectrum of the new compound **2**.

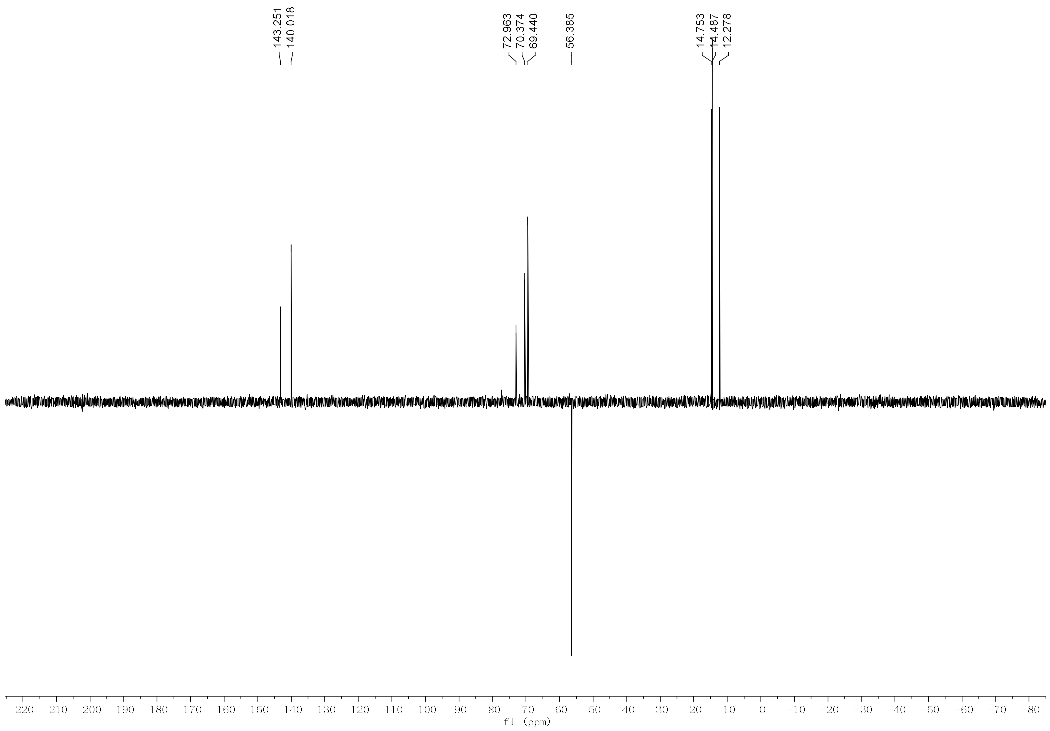

Figure S10. COSY NMR spectrum of the new compound **2** in $CDCl_3$.

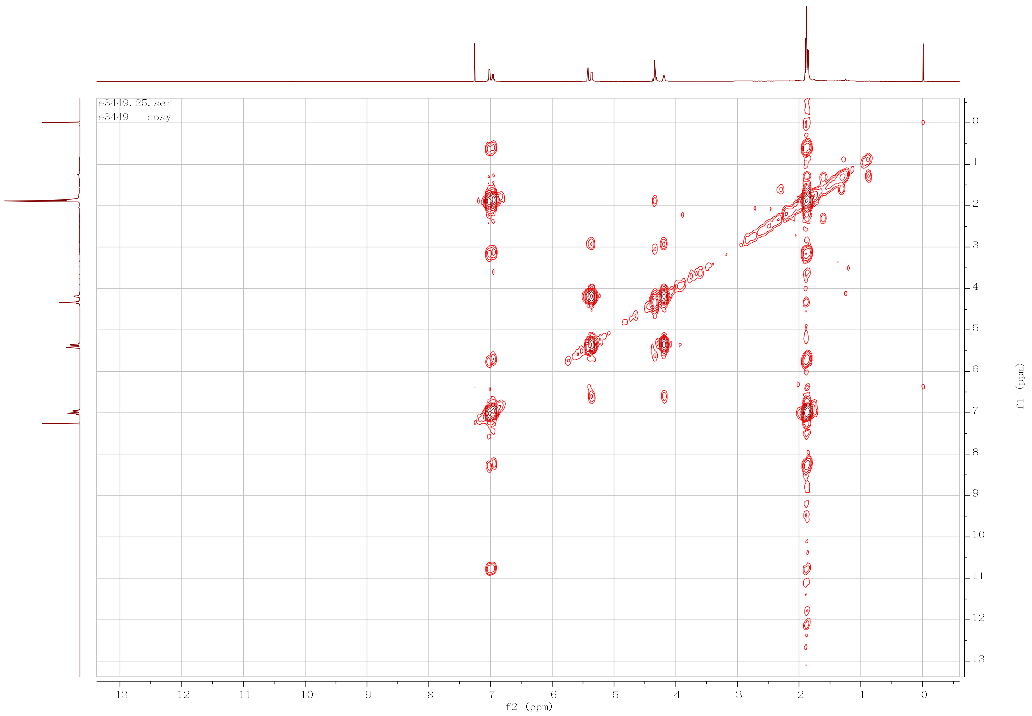

Figure S11. HSQC NMR spectrum of the new compound **2** in $CDCl_3$.

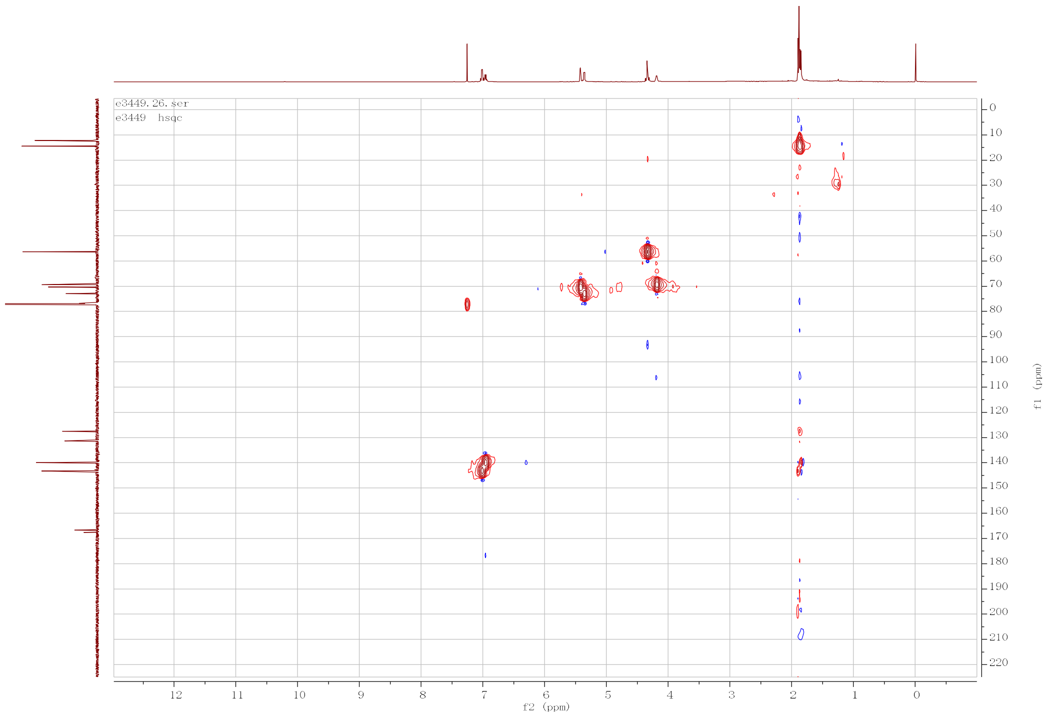

Figure S12. HMBC NMR spectrum of the new compound **2** in $CDCl_3$.

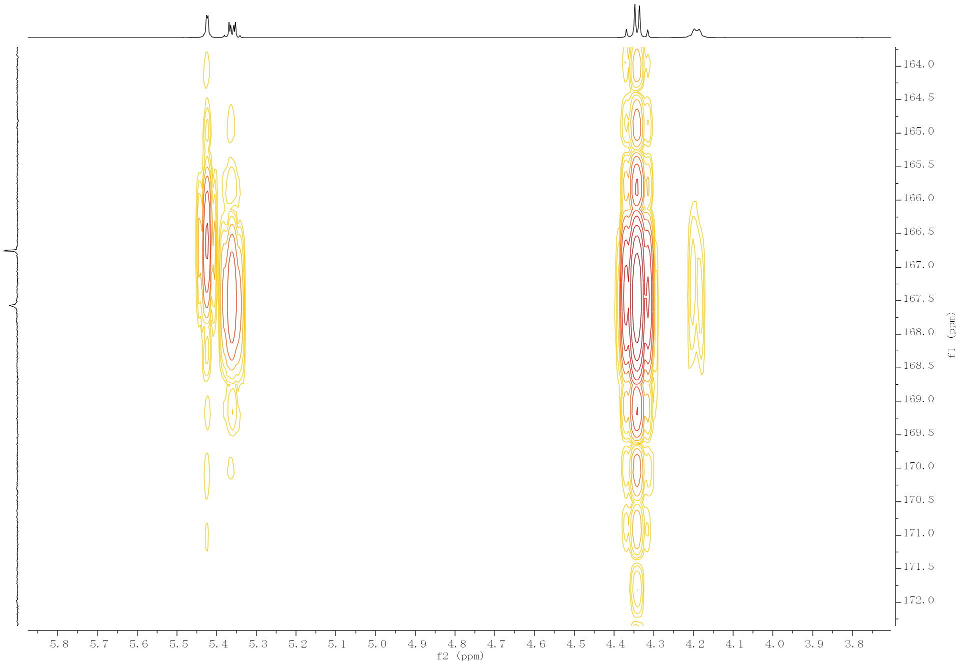

Figure S13. ROESY NMR spectrum of the new compound **2** in $CDCl_3$.

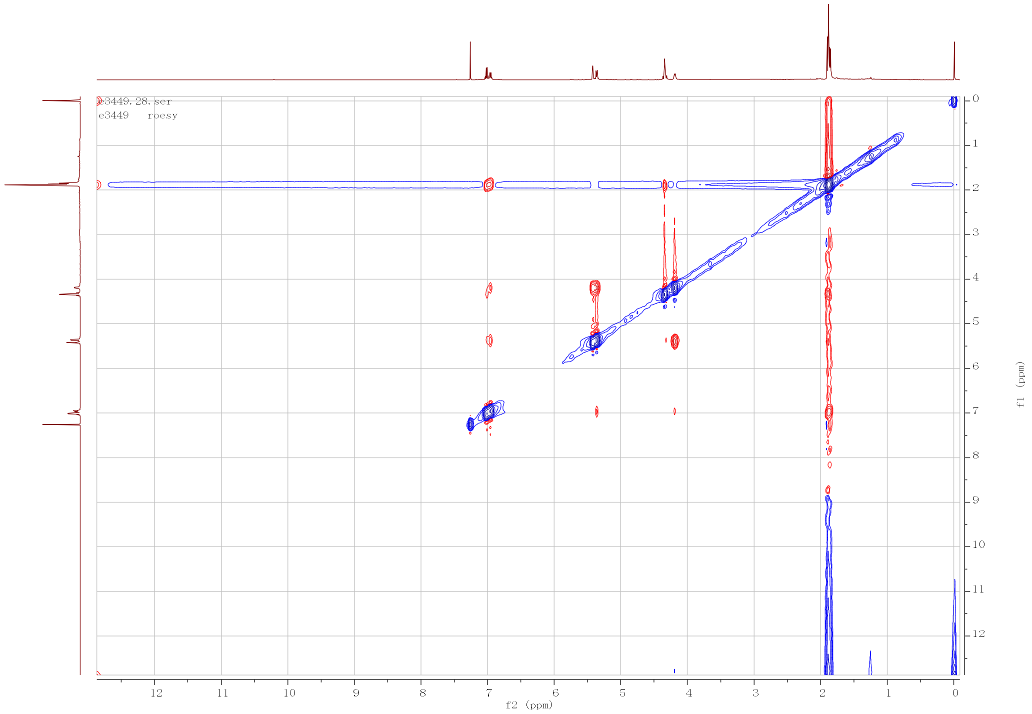

Figura S14. Mass spectra of **2**.

Qualitative Analysis Report

**Data Filename**	151014ESIA6.d	**Sample Name**	Esj193
**Sample Type**	Sample	**Position**	
**Instrument Name**	Agilent G6230 TOF MS	**User Name**	KIB
**Acq Method**	ESI.m	**Acquired Time**	10/13/2015 2:58:16 PM
**IRM Calibration Status**	Success	**DA Method**	ESI.m
**Comment**			

Sample Group **Info.**
Acquisition SW 6200 series TOF/6500 series
Version Q-TOF B.05.01 (B5125.2)

User Spectra

Fragmentor Voltage	Collision Energy	Ionization Mode
200	0	ESI

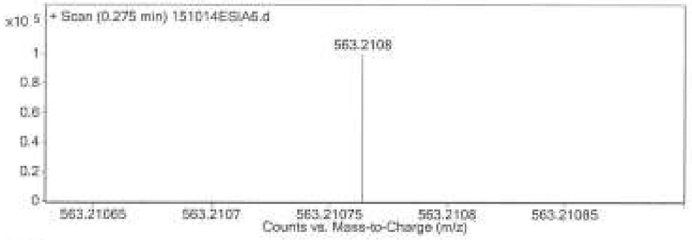

Peak List

m/z	z	Abund	Formula	Ion
121.0509	1	32932.61		
563.2108	1	100008.46	C26 H36 Na O12	M+
564.2135	1	23071.23	C26 H36 Na O12	M+
922.0098	1	43722.61		
1103.4313	1	128318.27		
1104.4338	1	66682.92		
1105.4364	1	21633.43		
1643.6494	1	49137.98		
1644.6521	1	40301.27		
1645.6555	1	18181.74		

Formula Calculator Element Limits

Element	Min	Max
C	0	200
H	0	400
O	8	15
Na	1	1

Formula Calculator Results

Formula	CalculatedMass	CalculatedMz	Mz	Diff. (mDa)	Diff. (ppm)	DBE
C26 H36 Na O12	563.2105	563.2099	563.2108	-0.7	-1.3	8.5000

--- End Of Report ---

Agilent Technologies Page 1 of 1 Printed at: 3:02 PM on: 10/13/2015

Figure S15. 1H NMR (400 MHz, CDCl3) spectrum of compound **3**.

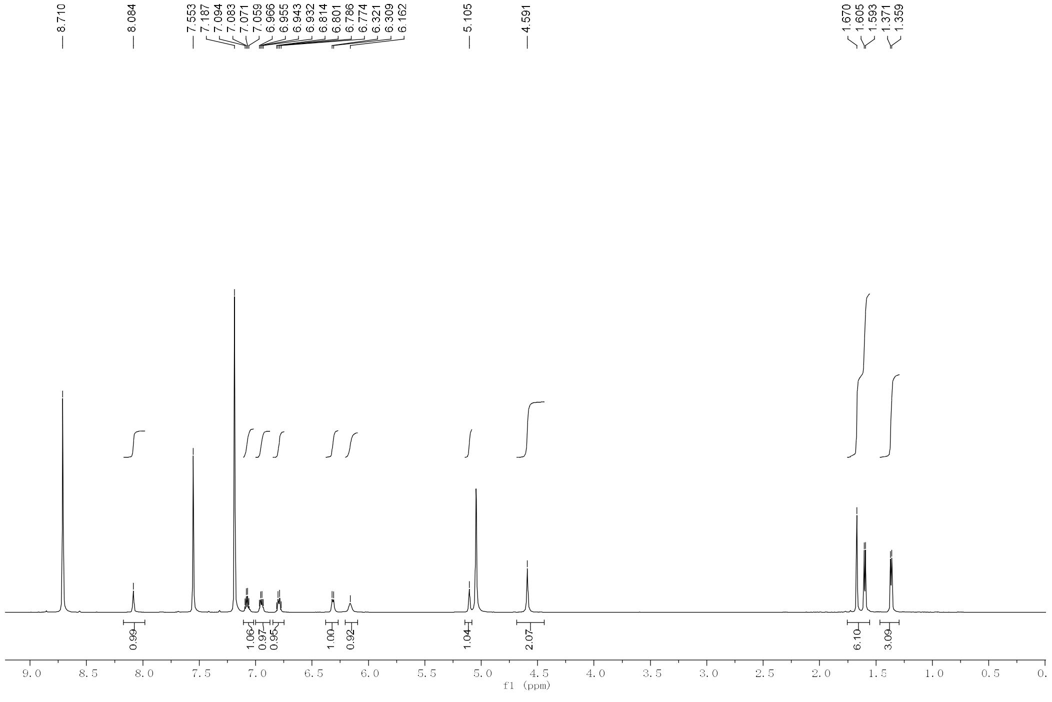

Figure S16. ^{13}C NMR (100 MHz, CDCl3) spectrum of the compound **3**.

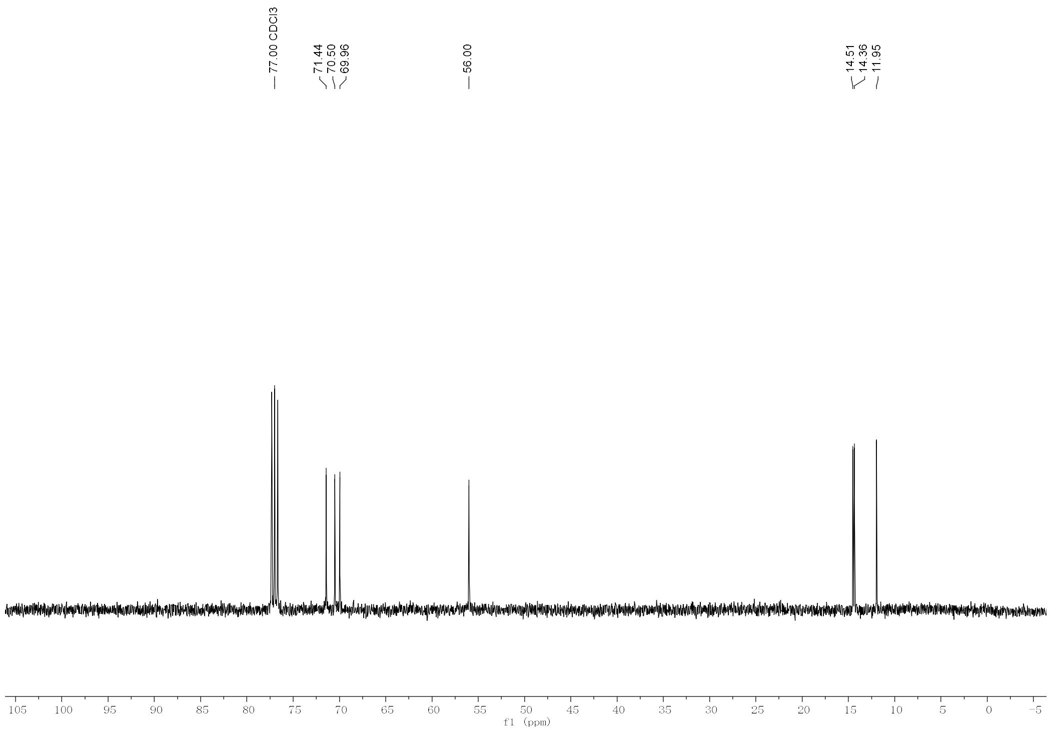

Figure S17. COSY NMR spectrum of the new compound **3** in $CDCl_3$.

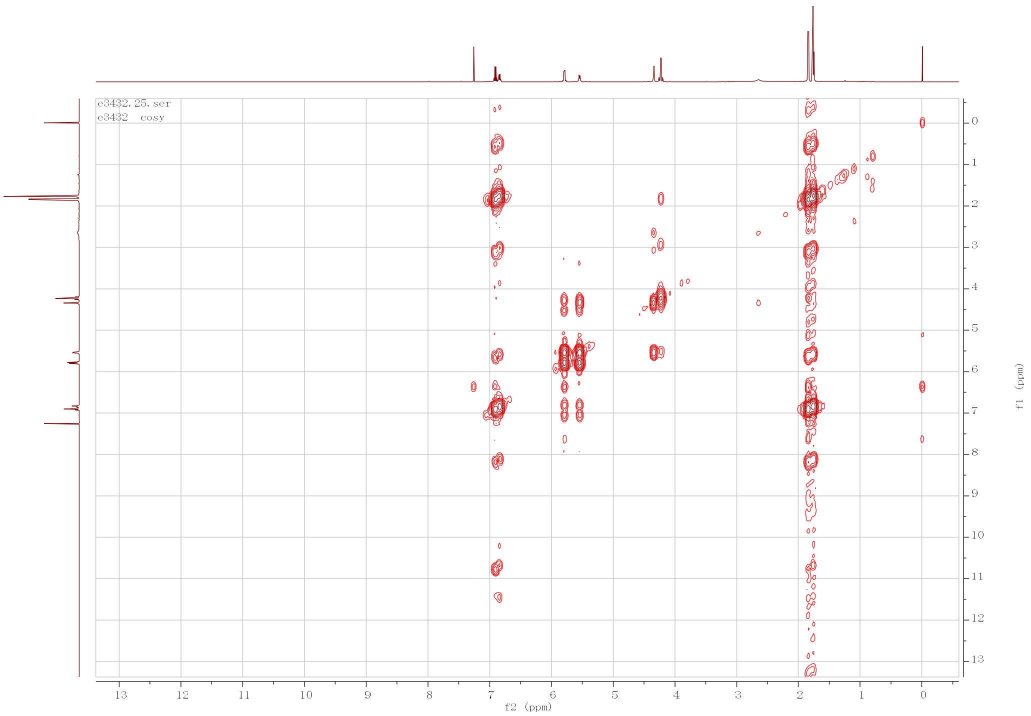

Figure S18. HSQC NMR spectrum of the new compound **3** in $CDCl_3$.

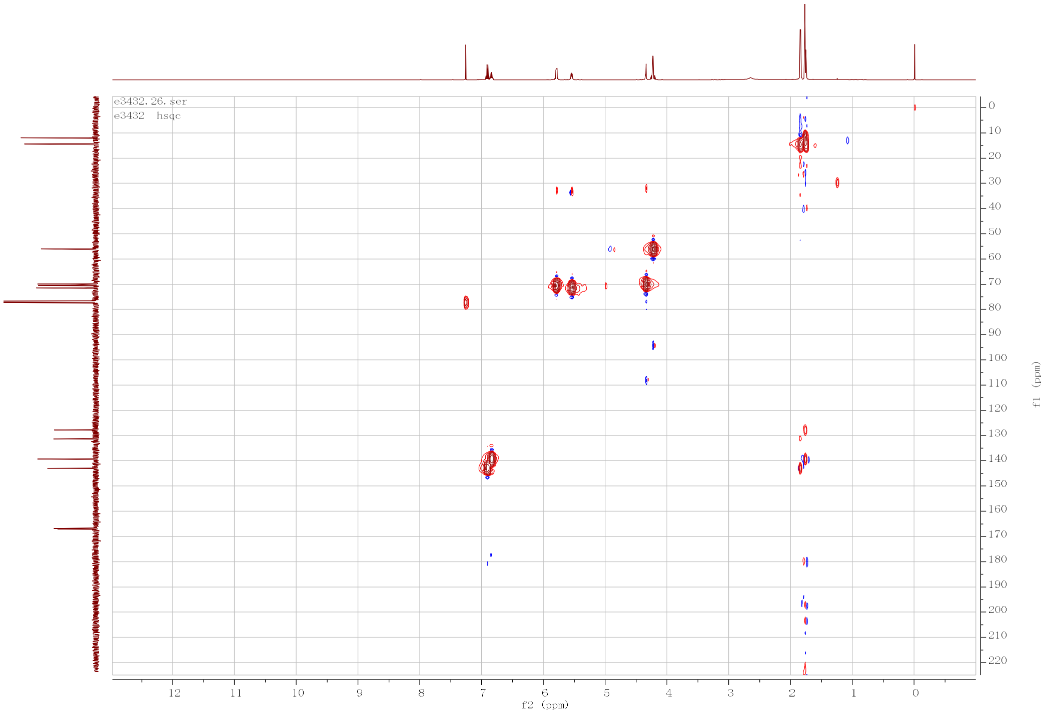

Figure S19. HMBC NMR spectrum of the new compound **3** in $CDCl_3$.

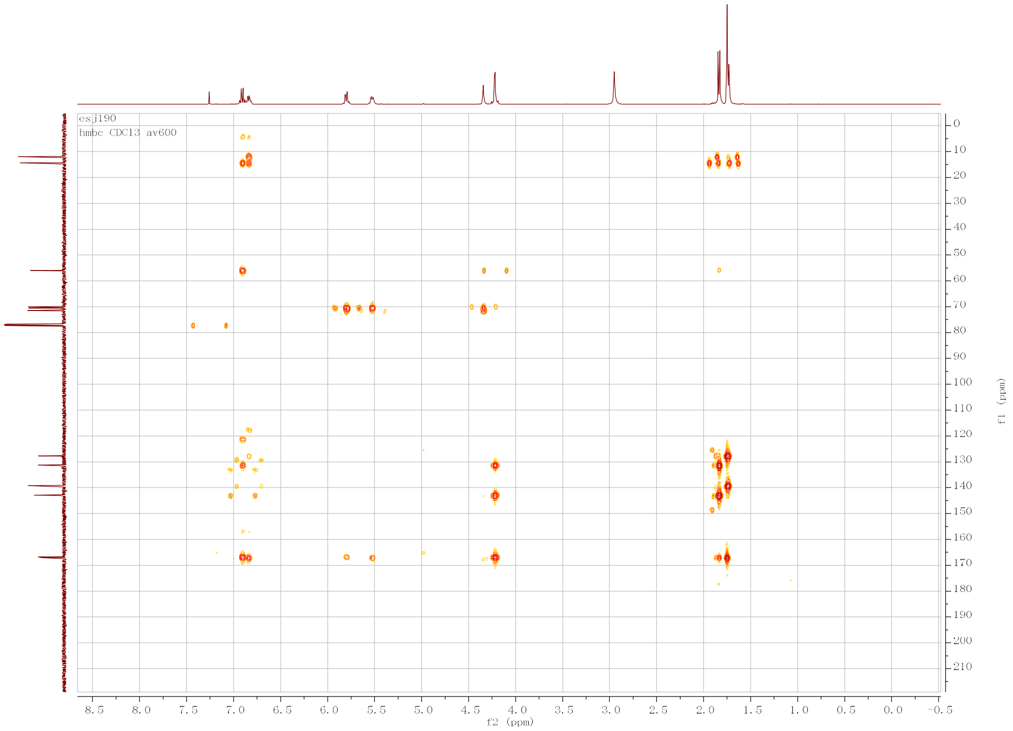

Figure S20. ROESY NMR spectrum of the new compound **3** in $CDCl_3$.

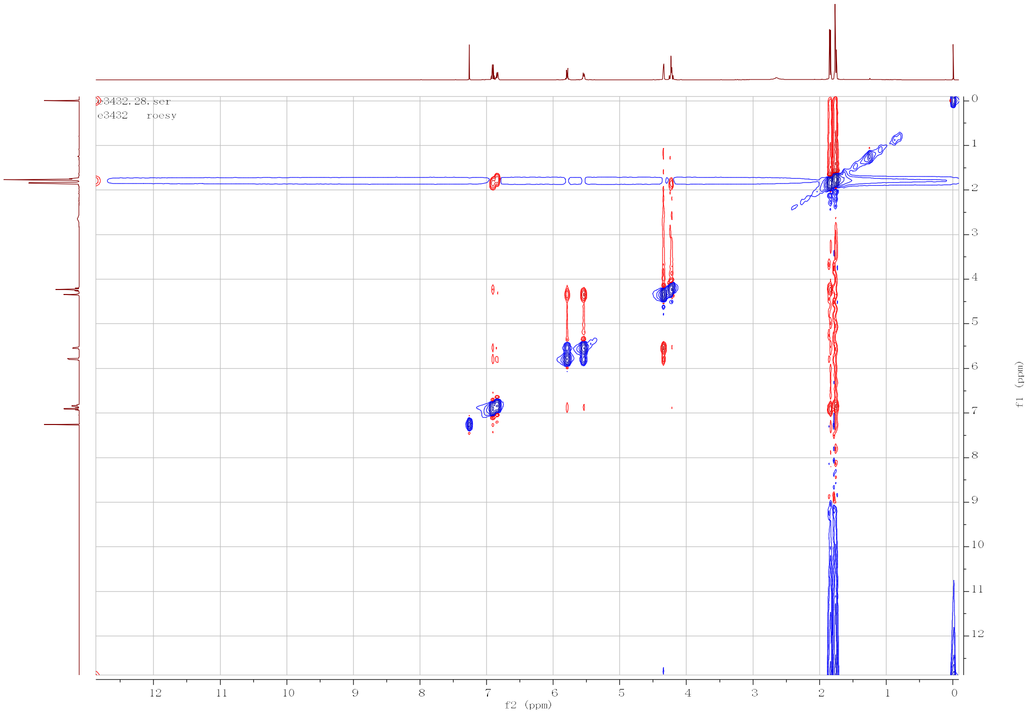

Figura S21. Mass spectra of **3**.

Qualitative Analysis Report

**Data Filename**	151014ESIA7.d	**Sample Name**	Esj190
**Sample Type**	Sample	**Position**	
**Instrument Name**	Agilent G6230 TOF MS	**User Name**	KIB
**Acq Method**	ESI.m	**Acquired Time**	10/13/2015 2:56:54 PM
**IRM Calibration Status**	Success	**DA Method**	ESI.m
**Comment**			

Sample Group **Info.**

Acquisition SW 6200 series TOF/6500 series

Version Q-TOF B.05.01 (B5125.2)

User Spectra

Fragmentor Voltage	Collision Energy	Ionization Mode
200	0	ESI

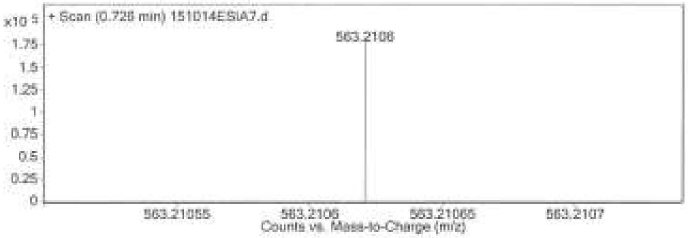

Peak List

m/z	z	Abund	Formula	Ion
121.0509	1	20890.8		
241.1698	1	21775.66		
563.2106	1	182976.83	C26 H36 Na O12	M+
564.2133	1	42337.3	C26 H36 Na O12	M+
922.0098	1	26542.91		
1098.4735	1	24148.25		
1099.476	1	13218.78		
1103.4311	1	279570.56		
1104.4341	1	152029.06		
1105.4369	1	52730.64		

Formula Calculator Element Limits

Element	Min	Max
C	0	200
H	0	400
O	8	15
Na	1	1

Formula Calculator Results

Formula	CalculatedMass	CalculatedMz	Mz	Diff. (mDa)	Diff. (ppm)	DBE
C26 H36 Na O12	563.2105	563.2099	563.2106	-0.6	-1.1	8.5000

--- End Of Report ---

Figure S22. ^{1}H NMR (400 MHz, CDCl3) spectrum of compound **4**.

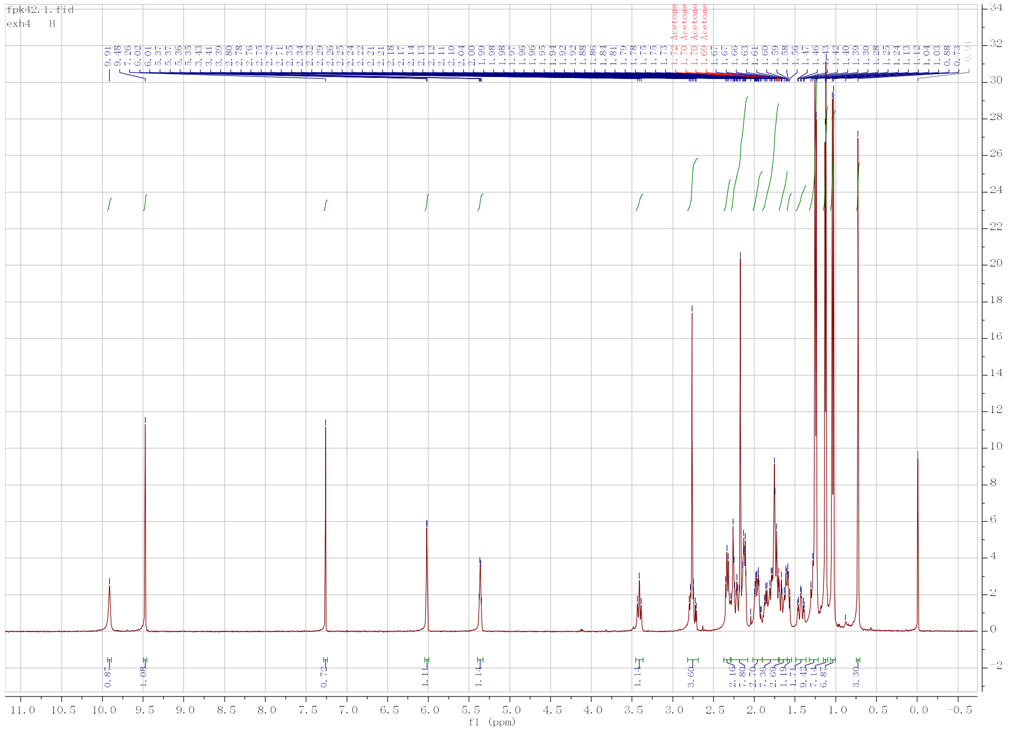

Figure S23. ^{13}C NMR (100 MHz, CDCl3) spectrum of the compound **4**.

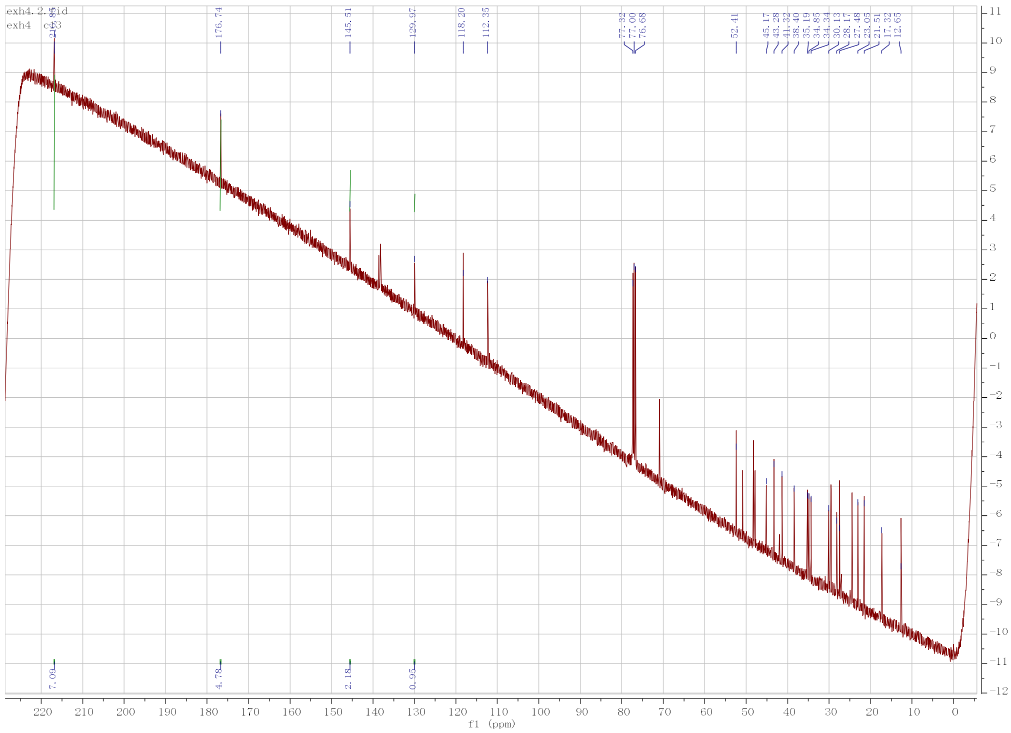

Figure S24. HSQC NMR spectrum of the new compound **4** in $CDCl_3$.

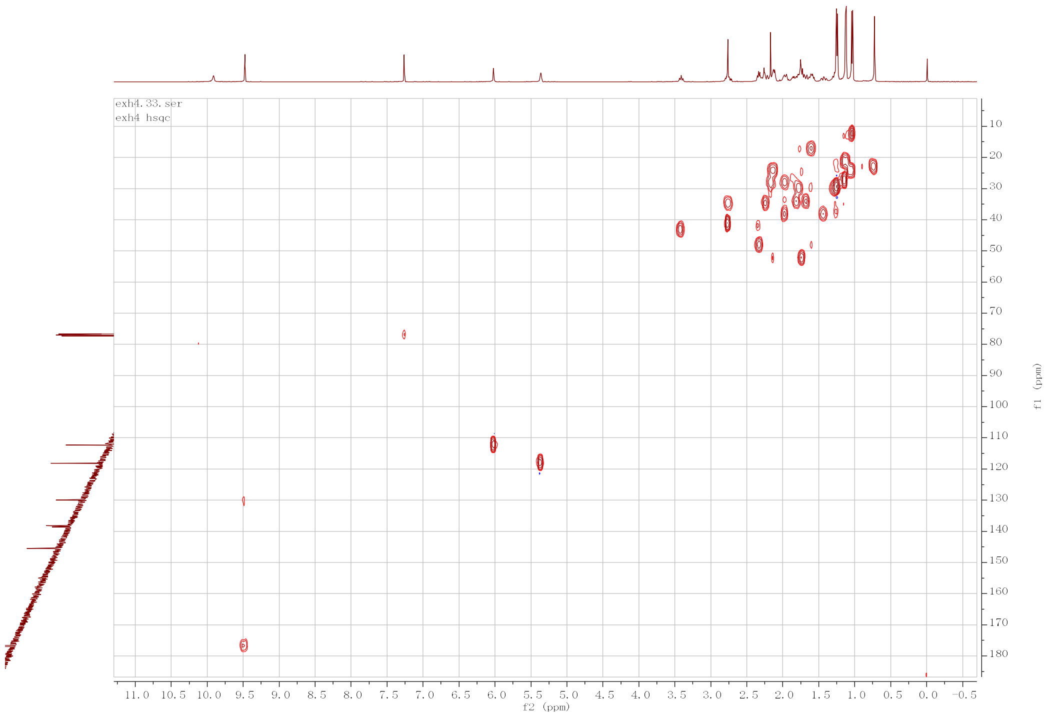

Figure S25. HMBC NMR spectrum of the new compound **4** in $CDCl_3$.

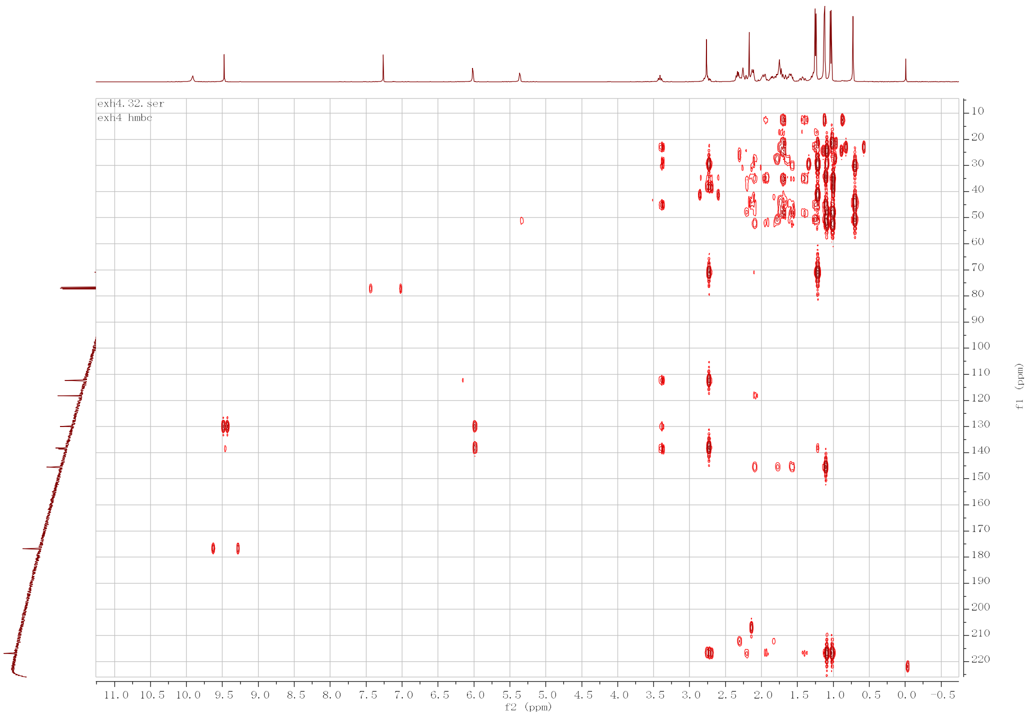

Figura S26. Mass spectra of **4**.

Qualitative Analysis Report

**Data Filename**	151020ESIA1.d	**Sample Name**	Exh4
**Sample Type**	Sample	**Position**	
**Instrument Name**	Agilent G6230 TOF MS	**User Name**	KIB
**Acq Method**	ESI.m	**Acquired Time**	10/20/2015 9:56:14 AM
**IRM Calibration Status**	Success	**DA Method**	ESI.m
**Comment**			

Sample Group **Info.**
Acquisition SW 6200 series TOF/6500 series
Version Q-TOF B.05.01 (B5125.2)

User Spectra

Fragmentor Voltage	Collision Energy	Ionization Mode
200	0	ESI

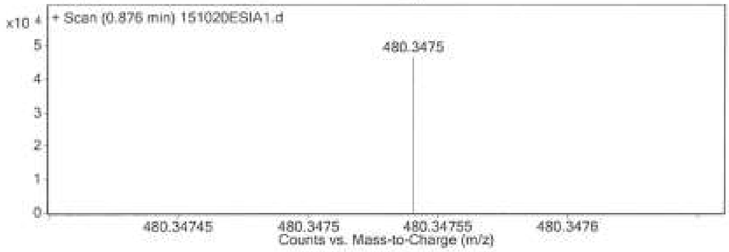

Peak List

m/z	z	Abund	Formula	Ion
121.0509	1	28187.4		
480.3475	1	46797.64	C31 H46 N O3	M+
481.3509	1	14439.68	C31 H46 N O3	M+
922.0098	1	50973.05		
923.0107	1	7334.34		
959.6868	1	13955.11		
960.6903	1	9346.11		
1461.0073	1	19270.99		
1462.0104	1	21027.77		
1463.0139	1	9299.57		

Formula Calculator Element Limits

Element	Min	Max
C	0	200
H	0	400
O	0	8
N	1	1

Formula Calculator Results

Formula	CalculatedMass	CalculatedMz	Mz	Diff. (mDa)	Diff. (ppm)	DBE
C31 H46 N O3	480.3478	480.3472	480.3475	-0.3	-0.6	9.5000

--- End Of Report ---

Agilent Technologies Page 1 of 1 Printed at: 10:08 AM on: 10/20/2015

Figure S27. ^{1}H NMR (400 MHz, CDCl3) spectrum of compound **5**.

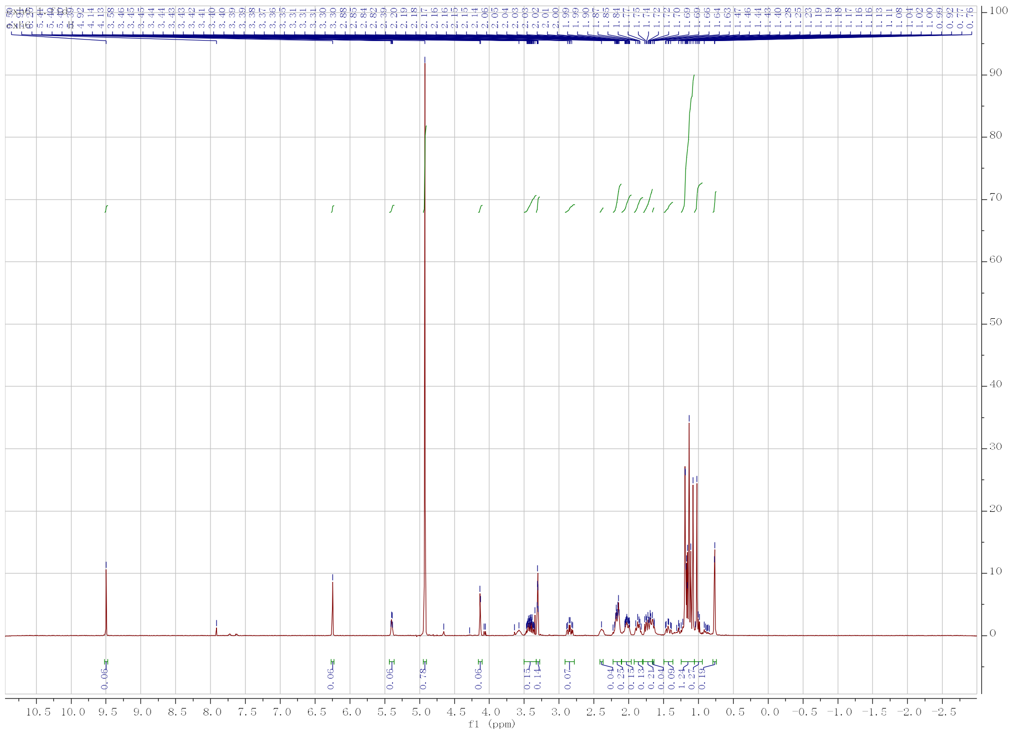

Figure S28. ^{13}C NMR (100 MHz, CDCl3) spectrum of the compound **5**.

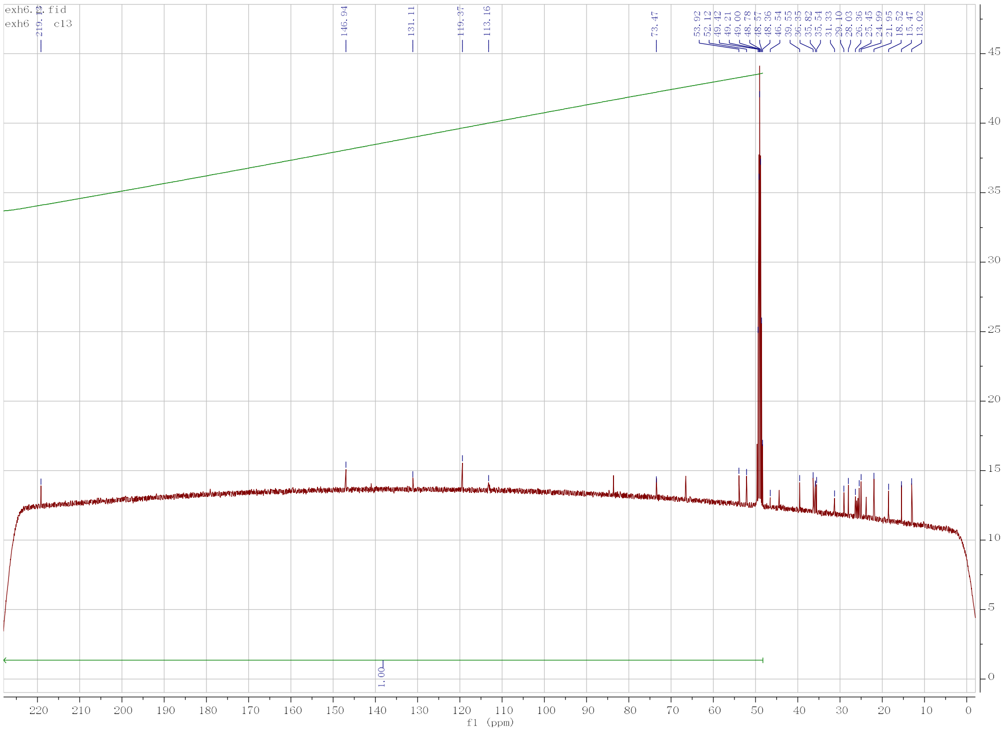

Figure S29. HSQC NMR spectrum of the new compound **5** in $CDCl_3$.

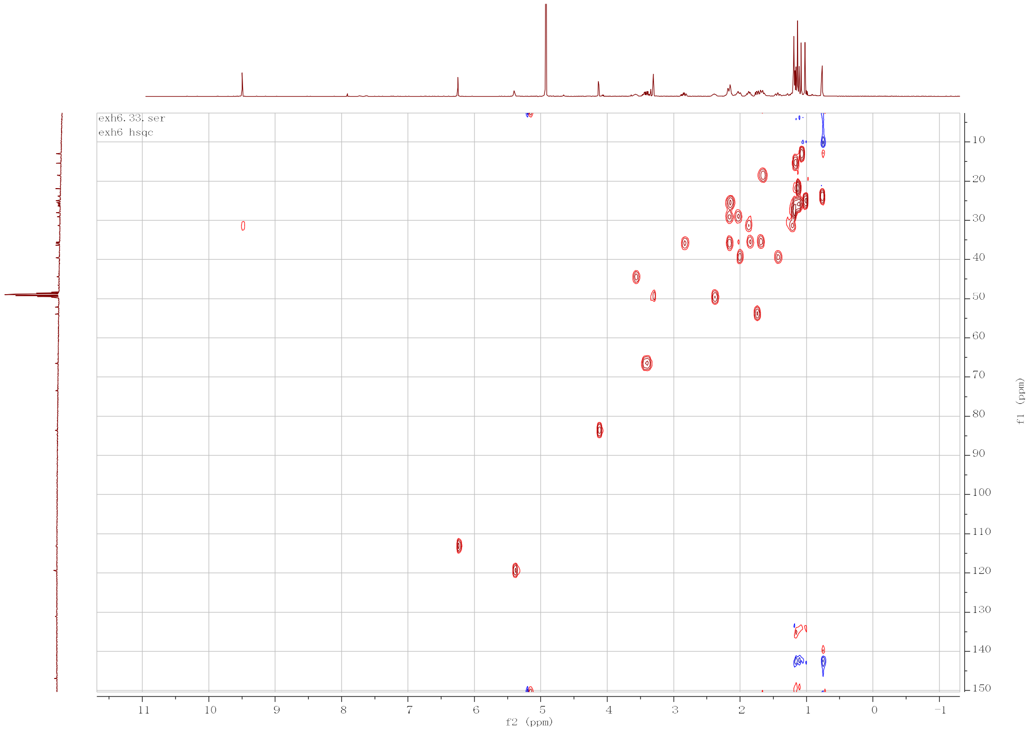

Figure S30. HMBC NMR spectrum of the new compound **5** in $CDCl_3$.

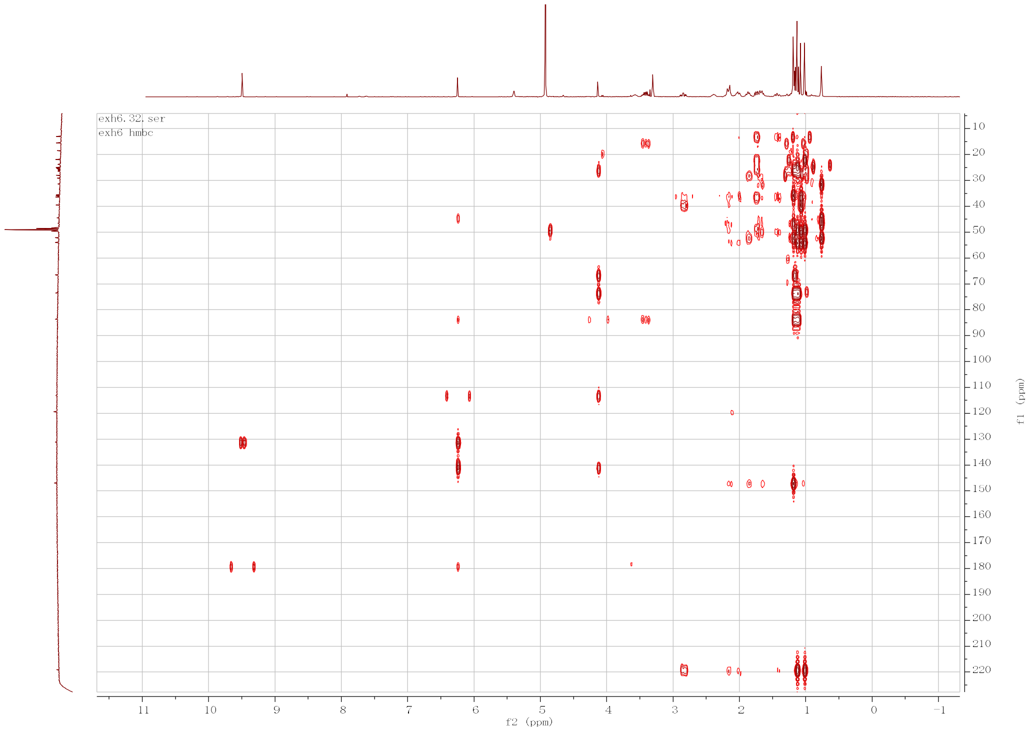

Figura S31. Mass spectra of **5**.

Qualitative Analysis Report

**Data Filename**	151020ESIA2.d	**Sample Name**	Exh6
**Sample Type**	Sample	**Position**	
**Instrument Name**	Agilent G6230 TOF MS	**User Name**	KIB
**Acq Method**	ESI.m	**Acquired Time**	10/20/2015 9:59:24 AM
**IRM Calibration Status**	Success	**DA Method**	ESI.m
**Comment**			

**Sample Group**		**Info.**
**Acquisition SW Version**	6200 series TOF/6500 series Q-TOF B.05.01 (B5125.2)	

User Spectra

Fragmentor Voltage	Collision Energy	Ionization Mode
200	0	ESI

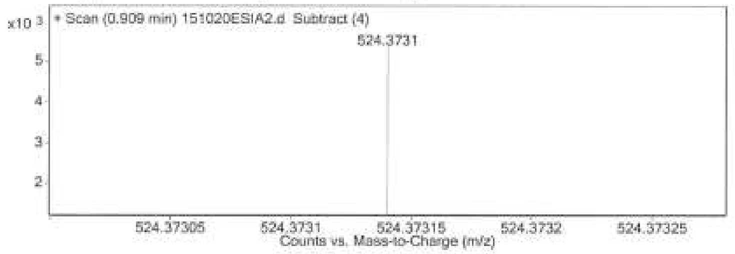

Peak List

m/z	z	Abund	Formula	Ion
524.3731	1	5387.93	C33 H50 N O4	M+
525.376	1	1591.48	C33 H50 N O4	M+
831.573	1	1321.41		
859.6045	1	1504.39		
1047.7324	1	2278.88		
1048.737	1	1410.99		
1069.7195	1	1366.65		
1593.0828	1	1807.55		
1594.0857	1	1774.6		
1595.0885	1	1161.32		

Formula Calculator Element Limits

Element	Min	Max
C	0	200
H	0	400
O	0	8
N	1	1

Formula Calculator Results

Formula	CalculatedMass	CalculatedMz	Mz	Diff. (mDa)	Diff. (ppm)	DBE
C33 H50 N O4	524.3740	524.3734	524.3731	0.4	0.7	9.5000

--- End Of Report ---

Figura S32. ECD spectrum of **3**.

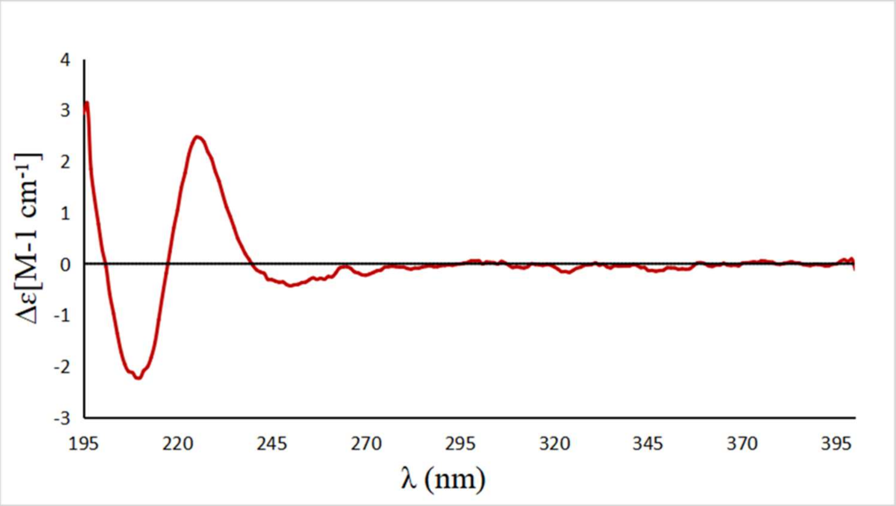

File: CD BBP03432-1mm(195-400)19070420.dsx

ProBinaryX

Attributes :

- Time Stamp :Thu Jul 04 17:41:32 2019

- File ID : {B6B4C4DE-D0D7-44b7-90AD-FAFA86D01D22}

- Is CFR Compliant : false

- Original data has not been modified.

Remarks:

- User: APLService

- Date: 2019/07/04
- Instrument: 0218
- DetectorType: PMT
- DichOS Calibration Correction Curve: 0218/1
- HV (CDDC channel): 0 v
- Time per point: 1 s
- Description: Sample 1
- Concentration: 0.0930mg/mL MeOH
- Pathlength: 1 mm
- Temperature: 20℃

Settings:

- Time-per-point: 1s (25us x 40000)
- SE
- Wavelength: 195nm - 400nm
- Step Size: 1nm
- Bandwidth: 1nm
